# Bridging the Gap: The Impact of Preoperative Nutritional Status and Inflammation in Postoperative Pain in Elderly Patients

**DOI:** 10.1155/anrp/6832202

**Published:** 2025-08-28

**Authors:** Rafail Ioannidis, Despoina Sarridou, Christina Tsigalou, Adamantios Bampoulas, Pelagia Chloropoulou

**Affiliations:** ^1^Anesthesiology and Pain Medicine Department, General Hospital of Drama, Drama, Greece; ^2^Anesthesiology and Intensive Care Department, Aristotle University of Thessaloniki, Thessaloniki, Greece; ^3^Medical Microbiology-Immunology Department, Democritus University of Thrace, Alexandroupoli, Greece; ^4^School of Computer Science, University College Dublin, Dublin, Ireland; ^5^Anesthesiology and Pain Medicine Department, Democritus University of Thrace, Alexandroupoli, Greece

**Keywords:** albumin, CRP, inflammation, MNA, NUTRIC, nutrition, pain, scores

## Abstract

Nutritional screening is gaining recognition in perioperative medicine, as anesthesiologists need to assess patients' nutritional status to identify malnutrition risks. Poor nutritional status is associated with increased perioperative complications, including postoperative pain. Effective pain management is crucial to prevent acute pain from developing into chronic pain. However, the link between malnutrition and pain is not well established, prompting interest in whether nutritional assessment tools correlate with pain severity. The Mini Nutritional Assessment Short-Form (MNA-SF) is a validated screening tool for geriatric patients, recommended by European Society for Clinical Nutrition and Metabolism (ESPEN) for routine use. The modified Nutrition Risk in Critically Ill (mNUTRIC) score evaluates critically ill patients' nutritional risk, guiding interventions to improve outcomes. This study aimed to explore the relationship between nutritional status, inflammatory markers, and postoperative pain in elderly surgical patients to optimize care. A prospective study involving 108 elderly patients (≥ 70 years) assessed the preoperative nutritional status using MNA-SF, mNUTRIC, Acute Physiology and Chronic Health Evaluation (APACHE), Sequential Organ Failure Assessment (SOFA), and inflammatory biomarkers (C-reactive protein (CRP) and albumin (Alb)). Postoperative pain was measured at surgery, 30 days, and 6 months. Statistical analysis found significant links between the nutritional status and pain outcomes. Higher mNUTRIC and APACHE scores correlated with increased pain, while higher Alb and MNA-SF scores were associated with lower pain levels. Chronic pain at 6 months was strongly linked to poor preoperative nutritional and inflammatory status. Findings suggest that nutritional deficiencies and inflammation are associated with postoperative pain and recovery. Integrating nutritional screening into preoperative assessments could improve outcomes by guiding interventions. Future research should refine predictive models to better understand the complex interplay between nutrition, inflammation, and pain in perioperative care.

**Trial Registration:** ClinicalTrials.gov identifier: NCT06802575

## 1. Introduction

One of the emerging trends in perioperative medicine is nutrition screening. Anesthesiologists should be aware of patients' nutritional status, recognize the risk of malnutrition, and be capable of screening nutritional status both preoperatively and postoperatively. However, the exact definitions of malnutrition have yet to be fully established [1].

Patient nutritional status is closely linked to various perioperative and postoperative complications [2]. Postoperative pain is one of the most significant concerns, not only for anesthesiologists but also for patients. Effective postoperative pain management aims to control pain following surgery for the best possible recovery of patients. It is crucial for anesthesiologists to manage pain and ensure that all patients remain pain free, not only to facilitate their recovery and transition back to daily life and activities but also to prevent acute postoperative pain from progressing into chronic pain [3].

Recent literature has not yet provided clear evidence on the relationship between malnutrition and acute or chronic pain [4, 5]. However, it is interesting to investigate whether the biomarkers that are suggested for detecting malnutrition, as well as the commonly used nutritional assessment scores, could be linked to higher acute and chronic pain scores [6, 7]. Malnutrition is increasingly seen as an important modifiable determinant in postoperative pain on a multifactorial pathophysiological basis. Malnourishment patients show a continuous low-grade inflammatory status with increased serum levels of specific cytokines like interleukin-6 (IL-6) and tumor necrosis factor-alpha (TNF-α) that sensitize peripheral nociceptive afferents and contribute to central sensitization, ultimately increasing perception of pain. Insufficient supplies of anti-inflammatory nutrients (omega-3 fatty acids, vitamin D, and zinc) are also responsible for inability to resolve inflammation and heal the tissues causing the prolongation of the pain-promoting part of the postsurgical recovery. In addition, malnutrition can render immune competence inadequate, with a higher incidence of postoperative complications and infectious diseases contributing to the enhancement of pain. In addition, low levels of key micronutrients such as the B vitamins and magnesium slow down nerve repair and add fuel to pain pathways. Taken together, these factors show that poor nutrition makes postsurgery pain both stronger and longer because it tips the body's pro- and anti-inflammatory balance, slows healing in tissues and nerves, and rewires the way pain messages are sent.

The Mini Nutritional Assessment (MNA) is a brief and reliable tool for nutritional screening in both independent and clinically relevant elderly populations [8, 9]. It includes questions tailored to geriatric patients regarding their nutrition, health status, independence, quality of life, cognition, mobility, and self-perceived health [10]. The European Society for Clinical Nutrition and Metabolism (ESPEN) recommends the MNA for routine geriatric evaluations [11]. To simplify its use, Rubenstein et al. developed the MNA Short-Form (MNA-SF), a six-question version that selects items from the full MNA based on their high sensitivity, specificity, and strong correlation with the complete assessment [12]. This shorter version classifies elderly individuals as either well-nourished or at risk of malnutrition, with the full MNA only required if the patient is deemed at risk. The MNA-SF demonstrates diagnostic accuracy comparable to the full MNA for identifying well-nourished individuals and offers a time-saving alternative [13, 14].

The Nutrition Risk in Critically Ill (NUTRIC) score, developed by Heyland et al. [15], is designed to identify critically ill patients who may benefit from intensive protein-energy therapy during their ICU stay [16, 17]. The NUTRIC score evaluates various baseline characteristics to determine how nutritional interventions affect ICU patients based on their initial risk [18–20]. Although the measurement of IL-6 is a component of the NUTRIC score, it is not routinely available in most ICUs. Therefore, IL-6 can be omitted from the score, resulting in an adjusted version known as the modified NUTRIC (mNUTRIC) score. The mNUTRIC score includes seven clinical and laboratory parameters: age, BMI, Acute Physiology and Chronic Health Evaluation (APACHE) II score, serum albumin (Alb), serum creatinine, serum sodium, and serum glucose. Studies have shown that the mNUTRIC score is a reliable indicator of mortality in critically ill patients, with scores of four or higher indicating a significantly increased risk of mortality. The mNUTRIC score helps identify patients at high risk for poor outcomes and assists in guiding decisions regarding nutritional support. It is recommended that clinicians use the mNUTRIC score to assess nutritional risk and guide nutritional decisions in critically ill patients [21, 22]. In addition, the mNUTRIC score can be applied during the perioperative period without any modifications.

The main objective of this noninterventional, observational, prospective study is to evaluate the relationship between specific biomarkers and nutritional screening tests and the level of postoperative pain, whether acute or chronic, in surgical elderly patients with or without nutritional deficiencies in order to optimize postoperative care, filling knowledge gaps, personalize healthcare, and contribute to improving both pain management and nutritional care in elderly surgical patients, enhancing their recovery and quality of life.

## 2. Materials and Methods

Ethics approval was anonymized. This noninterventional, observational, prospective study included 108 surgical elderly patients aged ≥ 70 years, regardless of their nutritional status, over a 4-year period from April 2020 to March 2024. All patients signed a detailed consent form, including also our hospital's mandatory consent form for obtaining anesthesia and surgical intervention and received detailed information about the study. Exclusion criteria included patients with psychiatric disorders, cancer patients, those undergoing corticosteroid therapy or chemotherapy, individuals undergoing cardiothoracic procedures, and patients with chronic inflammatory gastrointestinal diseases.

All patients included in the study received either subarachnoid or general anesthesia. Prior to the induction of anesthesia, full monitoring was applied to each patient, including electrocardiogram, pulse oximetry, and noninvasive arterial pressure measurement. When general anesthesia was required, bispectral index (BIS), nociception level (NOL) monitoring, and temperature measurement were also applied. For both subarachnoid and general anesthesia, the same anesthetic protocol was strictly followed, using the same type of drugs and always the same type of atraumatic 25G needle (Pencan).

Preoperatively, specific laboratory tests were conducted on each patient, including those utilized for the purposes of this study: C-reactive protein (CRP) and Alb. In addition, preoperative assessments were conducted using the Visual Analog Scale (VAS), MNA-SF, mNUTRIC score, Sequential Organ Failure Assessment (SOFA) score, and APACHE score for each patient. All patients received general or subarachnoid anesthesia for their surgical procedures, and the same anesthesia protocol was followed consistently for each case.

In the postoperative period, VAS scores were recorded to assess acute postoperative pain, pain at 30 days postsurgery, and potential chronic pain at 6 months.

### 2.1. Statistical Analysis

The analytical strategy adopted in this study encompassed a comprehensive statistical evaluation designed to investigate and quantify associations between clinical predictor variables and ordinal postoperative pain outcomes. The analysis was structured into two main stages.

Initially, associations between all pairs of predictor and outcome variables were assessed using Spearman's rank-order correlation coefficient, a nonparametric measure suitable for examining monotonic relationships without assuming normality or linearity of the underlying data distributions [23, 24]. Predictor variables were utilized directly as continuous or semicontinuous measures, while ordinal outcome variables were converted into numerical codes, preserving their inherent order. Statistical significance for these correlation coefficients was evaluated using corresponding *p* values derived from the Spearman correlation tests, applying a conventional alpha threshold of 0.05. The resulting correlation coefficients and their associated *p* values were visualized using heatmaps to facilitate interpretation and comparison across variables.

Subsequently, the statistical power associated with detecting these observed Spearman correlations was estimated through a simulation-based approach. Given the absence of robust analytical methods to calculate power for Spearman correlations directly, a Monte Carlo simulation method was employed, following recommendations in recent literature [25, 26]. For each pairwise correlation, simulated datasets were generated under a bivariate normal assumption reflecting the magnitude of the observed correlation. Each simulated dataset mirrored the sample size of the original data, and the proportion of simulated datasets yielding statistically significant results (*p* < 0.05) provided an empirical estimate of statistical power. A total of 1000 simulation replicates were used to ensure stable power estimates.

The results from the power analysis were systematically arranged into matrices and visualized using heatmaps, allowing clear identification of variable pairs with sufficient power to detect the observed correlations reliably. This approach facilitated an informed interpretation of the observed correlations, highlighting both robust associations and areas requiring greater statistical power, potentially through increased sample sizes or improved measurement precision in future studies.

## 3. Results

A total of 108 patients were included in the study. The analysis investigated associations among various input clinical variables (MNA-SF, mNUTRIC, APACHE, SOFA, CRP, and Alb) as well as their relationships with target postoperative pain outcomes (presurgery pain, acute postsurgery pain, pain at 30 days postsurgery, and chronic pain at 6 months). The strength of these associations was quantified using Spearman's rank-order correlation coefficient, with statistical significance determined by *p* values, and complemented by a simulation-based power analysis to ensure robustness.

Very strong and statistically significant correlations were observed among several input variables, highlighting substantial interdependence. Specifically, APACHE showed a very strong correlation with mNUTRIC (*r* = 0.84, *p* < 0.001, and power ≈ 1.0) and strong correlations with SOFA (*r* = 0.83, *p* < 0.001, and power ≈ 1.0) and Alb (*r* = −0.78, *p* < 0.001, and power ≈ 1.0). Alb also demonstrated a strong negative correlation with mNUTRIC (*r* = −0.75, *p* < 0.001, and power ≈ 1.0). CRP exhibited weak to moderate correlations with the other input variables, with correlation coefficients ranging between 0.34 and 0.52, all significant (*p* < 0.001) and with high statistical power (≥ 0.93).

When examining associations between input and target variables, clear patterns emerged. Presurgery pain was moderately correlated with APACHE (*r* = 0.57, *p* < 0.001, and power = 1.0), Alb (*r* = −0.51, *p* < 0.001, and power = 1.0), SOFA (*r* = 0.51, *p* < 0.001, and power ≈ 1.0), and mNUTRIC (*r* = 0.48, *p* < 0.001, and power ≈ 1.0). Acute postsurgery pain showed weak yet significant correlations with Alb (*r* = −0.40, *p* < 0.001, and power = 0.99) and APACHE (*r* = 0.34, *p* < 0.001, and power = 0.96), indicating reliable detection of these associations. However, some weaker associations, particularly those approaching the lower boundary of the “weak” classification, displayed power estimates below the conventional threshold of 80%, suggesting insufficient statistical power and, therefore, reduced confidence in these specific associations.

Pain at 30 days postsurgery exhibited strong correlations with nutritional status measures, including negative correlations with MNA-SF (*r* = −0.70, *p* < 0.001, and power = 1.0) and Alb (*r* = −0.64, *p* < 0.001, and power = 1.0). In addition, moderate to strong positive correlations were identified with APACHE (*r* = 0.60, *p* < 0.001, and power = 1.0) and mNUTRIC (*r* = 0.54, *p* < 0.001, and power = 1.0).

Chronic pain at 6 months demonstrated strong correlations with clinical severity indices APACHE (*r* = 0.79, *p* < 0.001, and power = 1.0), mNUTRIC (*r* = 0.77, *p* < 0.001, and power = 1.0), Alb (*r* = −0.70, *p* < 0.001, and power = 1.0), SOFA (*r* = 0.70, *p* < 0.001, and power = 1.0), and MNA-SF (*r* = −0.62, *p* < 0.001, and power = 1.0), indicating that worse nutritional and clinical status significantly predict higher chronic pain severity.

**Figure 1:**
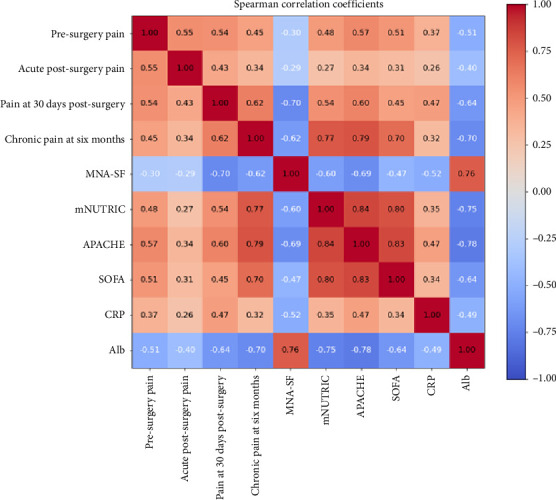
Spearman correlation coefficients.

**Figure 2:**
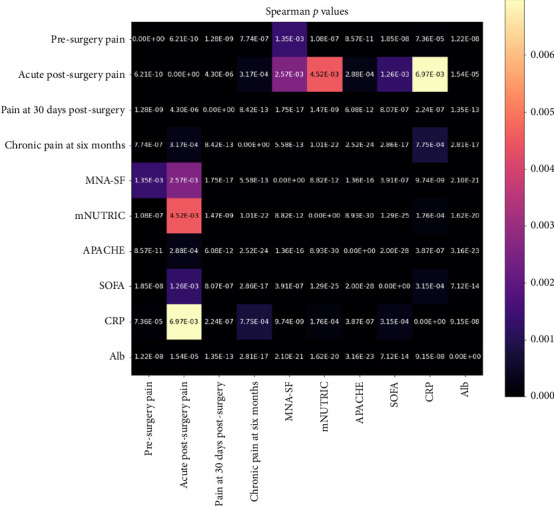
Spearman correlation *p* values.

**Figure 3:**
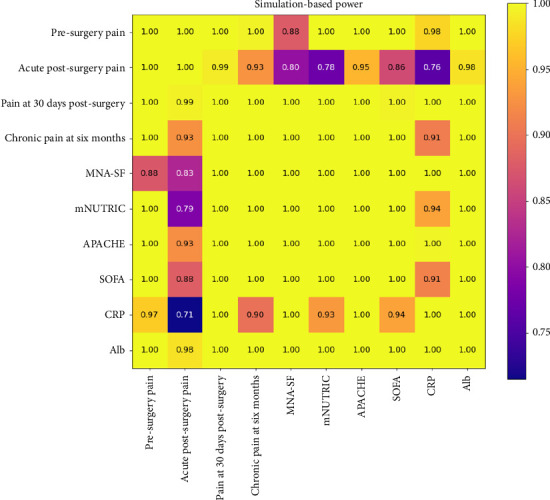
Simulation-based power of detecting each pairwise correlation among the four target and six input variables.

Three heatmaps are shown in Figures 1, 2, and 3 to illustrate the results of the correlation analysis. Each matrix is composed of 10 variables: the four target variables (presurgery pain, acute postsurgery pain, pain at 30 days postsurgery, and chronic pain at 6 months) and the six input variables (MNA-SF, mNUTRIC, APACHE, SOFA, CRP, and Alb).

In Figure 1, the Spearman correlation coefficients are visualized, with the color scale ranging from −1 (dark blue) to +1 (dark red). The direction and magnitude of each pairwise correlation are depicted by the hue and intensity of the colors.

In Figure 2, the *p* values (expressed in scientific notation) are presented. Darker cells correspond to smaller *p* values, signifying stronger statistical evidence against the null hypothesis of no association, while lighter cells indicate weaker evidence.

Finally, in Figure 3, the simulation-based power for detecting the pairwise correlations at *α* = 0.05 is displayed. Warmer colors (approaching yellow) indicate higher power, whereas cooler shades (moving toward magenta) reflect comparatively lower power.

## 4. Discussion

From a clinical perspective, rather than focusing on isolated features, the findings from the study suggest that the nutritional status, inflammation, and illness severity belong to a wider range of associated factors, which significantly affect the outcomes. For example, the correlation between pain at 30 days postsurgery and chronic pain at 6 months suggests that short-term complications may increase the risk of long-term pain, highlighting the requirement for comprehensive preoperative screening to evaluate multiple dimensions of patient health status.

It is very interesting that there are no further studies in medical database which tried to associate the nutritional status, inflammation, and acute and chronic postsurgical pain, and this is the reason that the results of this study are so unique. There are only limited data about the relationship of malnutrition and pain in specific pain syndromes, such as chronic musculoskeletal pain. Komolsuradej et al. used MNA-SF to prove the positive association between nutritional deficiencies and chronic musculoskeletal pain [27]. Elma et al.'s systematic review showcased the connection between eating habits and osteoarthritis and fibromyalgia [28].

When evaluating in this study postoperative outcomes at 30 days, the very low *p* values for APACHE, mNUTRIC, MNA-SF, and Alb, with either strong or moderate correlations with this category of pain, highlight their relevance. Likewise, in the assessment of chronic pain at 6 months postoperation, APACHE, SOFA, mNUTRIC, Alb, and MNA-SF emphasize their importance as potential indicators of chronic pain at 6 months postoperation because of the very low *p* values along with either strong or moderate correlations with this category of pain.

APACHE and SOFA scores are severity of illness classification and mortality prediction scores validated from numerous studies, which also appeared as relevant parameters to evaluate postsurgical pain. Furthermore, these scores play a connecting role in the relationship of severity illness, pain and malnutrition [29, 30].

A quite intriguing result of this study is that none of the input variables demonstrated excessive statistical strength or clinical relevance for short-term outcomes. This could be explained by the fact that the anesthetic agents provided always ensured adequate acute postoperative analgesia, which is a key aspect of anesthesiology. In addition, the role of the anesthesiologist in perioperative nutritional support is significant, as described in recent studies. This further emphasizes the importance of this study and the need to select key preoperative factors for assessing nutritional status [31].

For postoperative outcomes at 30 days and chronic outcomes associated with pain, MNA-SF and Alb emerged as the strongest correlated features, confirming their role as risk factors associated with nutritional and inflammatory deficiencies [10, 17].

The preoperative laboratory tests showed a strong correlation with nutritional screening assessments. These features primarily act on classic inflammatory markers, and the study emphasizes the significant link between malnutrition and inflammation. Specifically, an inverse relationship was found between the inflammation marker CRP and the MNA-SF, suggesting that poor nutritional status may reduce systemic inflammation. Furthermore, MNA-SF, APACHE, and Alb levels might reflect overlapping factors like metabolic reserve, nutritional health, and disease severity, which complicates isolating their individual effects, thereby reinforcing the connection between nutritional deficiencies and inflammation. Literature supports the idea that both malnutrition and inflammation can lower Alb levels by reducing its synthesis rate, with inflammation alone being linked to a higher fractional catabolic rate and, in extreme cases, an increased transfer of Alb from the vascular space. This leads to a vicious cycle where inflammation causes anorexia, impairs the effective use of protein and energy intake, and increases the breakdown of Alb. Moreover, it is notable that the negative correlation between Alb and CRP could be used as a screening tool for inflammation, guiding therapeutic interventions and preventing excessive correction of inflammatory patients [32–34].

The consistently high statistical power across most analyses provides confidence in the robustness and reliability of these observed relationships. Nevertheless, variable pairs demonstrating power below the 80% threshold highlight the need for cautious interpretation and suggest potential limitations due to the sample size or effect size. These results underscore the clinical importance of preoperative nutritional and inflammatory status, as well as overall clinical severity, as critical predictors of postoperative pain outcomes. Given the strong interdependencies among input variables, future studies employing multivariate analytical approaches with larger sample sizes may further clarify independent predictive relationships and enhance power for weaker associations.

The link between poor nutrition and pain stretches far beyond the hours spent in a surgical ward, weaving together missing vitamins, a shaky immune system, and a nervous system that misreads signals across different kinds of patients. Because surgery dumps a rush of stress and inflammation into the body, these patients provide a clear stage to watch how low nutrient levels can worsen pain; yet the same pattern shows up in people who never see an operating room. Malnutrition itself keeps slipping through the cracks, especially among older adults, people fighting long-term illness, and anyone living on a tight budget. Sadly, the same groups bear much of the burden of chronic pain, from achy knees in osteoarthritis to the stabbing or burning of neuropathic pain and the wide-body tenderness of fibromyalgia. Research hints that missing key nutrients—hands-on vitamin D, magnesium, and B vitamins—can turn up the volume on pain and dull the body's own pain-relief knobs in these individuals [35, 36]. Vitamin D gets the loudest applause: studies watching patients with low levels link such deficiency to ongoing muscle and joint ache as well as fatigue, and a small boost from supplements sometimes eases those complaints [37].

Chronic inflammation represents a further significant mechanism for the association of malnutrition with pain in nonsurgical settings. Protein-energy malnutrition, even of its subclinical form, is associated with the presence of elevated levels of proinflammatory cytokines such as TNF-α and IL-6, which play central roles in the pathophysiology of both nociceptive and neuropathic pain [38]. Malnourished patients may also demonstrate impaired resolution of inflammation due to reduced synthesis of anti-inflammatory mediators, thereby prolonging pain states and increasing the likelihood of central sensitization [39]. Pain and malnutrition frequently co-occur in cancer patients and work synergistically to compromise the quality of life. Cancer cachexia also involves systemic inflammation, weight loss, muscle wasting, and anorexia—characteristics that have a direct correlation with increased pain perception and reduced tolerance to pharmacologic analgesia [40]. Nutritional interventions in these patients are also able to have a dual effect of lowering inflammation and enhancing response to analgesics. For example, omega-3 fatty acids were found to decrease inflammatory cytokines and alleviate cancer pain although more potent trials are required to confirm these effects in nonsurgical settings [41].

From a neurological perspective, multiple micronutrients crucial to nerve health and neurotransmitter production that are found to be deficient in malnourished populations (B12, folate, and Mg) have also been linked to the development of peripheral neuropathy and increased pain perception [42]. This is of particular importance in patient groups such as those with diabetes mellitus, chronic alcoholism, or gastrointestinal malabsorptive disorders in whom neuropathic pain is a common and disabling symptom. In addition, the indirect influence of pain can be worsened by malnutrition due to effects on mood, sleep, and physical function. People who struggle with bad nutrition often battle depression and anxiety, and research shows that these mood issues can make pain hurt more and last longer [43]. On top of that, a poor diet tends to rob the body of good sleep, turning up the volume on pain signals and dulling the systems that usually calm them down [44].

The present investigation was intentionally designed as a noninterventional, prospective observational study. Consequently, the relationships we report—however strong or consistent—represent associations only and cannot establish causality, in line with the STROBE guidance for observational research [45]. Several threats to causal inference apply. (i) Residual confounding: unmeasured factors such as psychosocial stress or unrecorded comorbidities could influence both nutritional markers and pain trajectories [46]. (ii) Reverse or bidirectional relationships: malnutrition may exacerbate pain, but persistent pain can also suppress appetite and worsen nutritional status, as suggested by systematic evidence linking preoperative nutrition to postoperative outcomes [47]. (iii) Spurious correlations: purely statistical associations can arise from common upstream determinants that cannot be ruled out without experimental control [45]. Although multivariable adjustment and adequate power strengthen our findings, these measures mitigate rather than eliminate such concerns. Future work should, therefore, employ longitudinal causal-modeling techniques or randomized perioperative interventions—for example, omega-3-based immunonutrition or structured nutritional counseling, both of which have already shown reductions in postoperative pain and inflammatory markers [48]—to test whether altering these modifiable factors truly improves outcomes.

Regarding some limitations, although Alb is strongly related to nutritional status, it is affected by various non nutritional factors and its specificity and sensitivity are limited. For example, short-term nutritional changes (such as dietary adjustments within a few days) are difficult to reflect through Alb levels; abnormal liver function (such as hepatitis and cirrhosis), kidney disease (such as increased loss due to proteinuria), and severe infection or inflammation (inhibition of inflammatory cytokine synthesis) can significantly affect Alb levels and the decrease in Alb may not be related to nutrition. It is more likely a biomarker of “body reserves and stress state” rather than a direct nutritional indicator. Also, the APACHE and SOFA scoring systems are not specialized tools for assessing the nutritional status. The more severe the disease (high APACHE/SOFA score), the poorer the nutritional status (related to the nutritional score) and the more severe the disease itself may directly lead to increased pain (independently affecting pain).

## 5. Conclusion

The increasing recognition is that the nutritional status is a key determinant of postoperative recovery in elderly surgical patients, prompting the requirement for further investigation. Current literature and published research outcomes have already highlighted the role that nutrition plays in the total care and outcomes of critically ill patients. However, a perhaps more specific relationship between nutritional deficiencies and postoperative pain status remains inadequately explored. This study aimed to bridge this research gap by evaluating the predictive value of nutritional screening tools for acute and chronic postoperative pain, recovery, and long-term outcomes. MNA-SF consistently emerged as a key factor, associated with complications and pain, while mNUTRIC acted as a high-risk indicator linked to poorer recovery trajectories. Traditional severity scores, such as APACHE and SOFA, despite their correlations, provided limited evaluating power when adjusting for nutrition and inflammation, reflecting potential multicollinearity among variables. However, it is very important not to forget them in the preoperative nutritional assessment.

These findings support the integration of nutritional assessments into preoperative evaluations to identify at-risk patients and guide targeted interventions. Addressing nutritional and inflammatory deficiencies may reduce complications, promote faster recovery, and improve long-term quality of life.

The study limits its applicability to the general elderly surgical population and includes various types of surgical procedures from different surgical subspecialties. Also, the protocol of this study did not include measurement of inflammation markers postoperatively. Future research should focus on multicollinearity diagnostics, nonlinear modeling, and interaction terms to refine evaluating models and enhance their clinical applicability. Also, it would be enchanting to create a prediction model for postoperative pain based on the factors included in this study. More studies are needed in order to further investigate the relationship between the nutritional status, inflammatory markers, and postoperative pain in the perioperative setting.

## Data Availability

Data are available on request due to privacy/ethical restrictions.
